# Effects of Sr-HT-Gahnite on osteogenesis and angiogenesis by adipose derived stem cells for critical-sized calvarial defect repair

**DOI:** 10.1038/srep41135

**Published:** 2017-01-20

**Authors:** Guifang Wang, Seyed-Iman Roohani-Esfahani, Wenjie Zhang, Kaige Lv, Guangzheng Yang, Xun Ding, Derong Zou, Daxiang Cui, Hala zreiqat, Xinquan Jiang

**Affiliations:** 1 Department of Prosthodontics, Ninth People’s Hospital affiliated to Shanghai Jiao Tong University, School of Medicine, 639 Zhizaoju Road, Shanghai 200011, China; 2 Oral Bioengineering Lab, Shanghai Research Institute of Stomatology, Ninth People’s Hospital Affiliated to Shanghai Jiao Tong University, School of Medicine, Shanghai Key Laboratory of Stomatology, 639 Zhizaoju Road, Shanghai 200011, China; 3 Biomaterials and Tissue Engineering Research Unit, School of AMME, The University of Sydney, Sydney, NSW 2006, Australia; 4 Department of Stomatology, Shanghai Jiao Tong University Affiliated Sixth People’s Hospital, 600 Yishan Road, Shanghai 200233, China; 5 Institute of Nano Biomedicine and Engineering, Key Laboratory of Thin Film and Microfabrication Technology of Ministry of Education, Department of Instrument Science and Engineering, School of Electronic Information and Electrical Engineering, National Center for Translational Medicine, Collaborative Innovational Center for System Biology, Shanghai Jiao Tong University, 800 Dongchuan Road, Shanghai 200240, China

## Abstract

Tissue engineering strategies to construct vascularized bone grafts are now attracting much attention. Strontium-hardystonite-Gahnite (Sr-HT-Gahnite) is a strong, highly porous, and biocompatible calcium silicate based bio-ceramic that contains strontium and zinc ions. Adipose derived stem cells (ASCs) have been demonstrated to have the ability in promoting osteogenesis and angiogenesis. In this study, the effects of Sr-HT-Gahnite on cell morphology, cell proliferation, and osteogenic differentiation of ASCs were systematically investigated. The cell proliferation, migration and angiogenic differentiation of human umbilical vein endothelial cell (HUVECs) were studied. Beta-tricalcium phosphate/hydroxyapatite (TCP/HA) bioceramic scaffolds were set as the control biomaterial. Both bio-ceramics exhibited no adverse influence on cell viability. The Sr-HT-Gahnite scaffolds promoted cell attachment and alkaline phosphatase (ALP) activity of ASCs. The Sr-HT-Gahnite dissolution products enhanced ALP activity, matrix mineralization, and angiogenic differentiation of ASCs. They could also improve cell proliferation, migration, and angiogenic differentiation of HUVECs. Levels of *in vivo* bone formation with Sr-HT Gahnite were significantly higher than that for TCP/HA. The combination of Sr-HT-Gahnite and ASCs promoted both osteogenesis and angiogenesis *in vivo* study, compared to Sr-HT-Gahnite and TCP/HA bio-ceramics when administered alone, suggesting Sr-HT-Gahnite can act as a carrier for ASCs for construction of vascularized tissue-engineered bone.

Large (or ‘critical-sized’) bone defects, which cannot heal without intervention, arise from traumatic injury, infection and tumour resection, and present a major challenge in orthopaedic medicine. Current treatments using autografts or allografts have serious limitations due to second site surgery, donor site morbidity, limited availability, and limited integration with native bone. More importantly, the healing of large bone defects remains challenging using standard bone grafting procedures.

A tissue-engineered graft composed of bioactive scaffolds with mesenchymal stem cells would be a promising bone graft for critical size bone defects^1^. During ossification and bone repair process, the newly formed blood vessels are important for providing oxygen, nutrients, cytokines and growth factors. The close correlation between bone formation and vascularization is referred to as ‘angiogenic-osteogenic coupling’^2^. However, inducing vascular ingrowth during bone regeneration remains a challenge.

Efforts have been directed towards incorporating pro-angiogenic factors into tissue-engineered constructs in order to enhance their angiogenic potential^3^,^4^,^5^,^6^,^7^. Evidence emerged in recent studies that bioactive glasses/ceramics or their dissolution products not only enhance new bone formation but also could stimulate the expression of angiogenesis related genes. The potential for angiogenic induction by calcium silica-based materials suggests that it could be an alternative to inductive growth factors^8^,^9^,^10^,^11^. For their excellent osteogenic activity, calcium silicate based materials have been investigated for bone regeneration^12^. They are able to stimulate the osteogenic differentiation of stem and progenitor cells^13^. Calcium (Ca) and silica (Si) elements have been known to promote osteoblasts proliferation and differentiation, as well as stimulate the pro-angiogenesis of HUVECs^14^. Strontium (Sr) ions have been confirmed to stimulate bone formation and decrease bone resorption both *in vitro* and *in vivo* studies^15^,^16^. In addition, they increase the proliferation and migration of endothelial cells suggesting their positive role in osteogenesis and pro-angiogenesis^17^. Incorporation of Sr into calcium-silica bio-ceramic induced superior osteoinductivity and angiogenesis compared with a calcium-silica bio-ceramic^18^. We have recently developed a multi-component Sr and zinc (Zn) containing ceramic, Strontium-hardystonite-Gahnite (Sr-Ca_2_ZnSi_2_O_7_-ZnAl_2_O_4_), hereafter called Sr-HT Gahnite. Its mechanical properties closely match those of cancellous bone (compressive strength of 6 MPa^19^,^20^. Moreover, 3D printed Sr-HT Gahnite scaffolds showed a mechanical strength comparable to cortical bone that make them a potential candidate for treatment of segmental bone defects^21^.

Strontium and zinc ions released by this ceramic are known to promote bone regeneration. The implantation of Sr-HT-Gahnite scaffolds in radial defect model in the rabbits exhibited better new bone formation, compared to that obtained with TCP/HA scaffolds. Sr-HT-Gahnite promotion of osteogenesis may be attributable to the presence of Sr and Si. Sr-HT Gahnite scaffolds may indeed be the type of construct that induces vascularized tissue-engineered bone.

Another important element for bone engineering is the mesenchymal stem cells. It plays a important role in the successful restoration of tissue morphology and function^22^. As one of the most commonly used type of adult mesenchymal stem cells, ASCs are known to possess good bone regenerative^23^ and angiogenic^24^ capacity. ASCs exhibit a pericyte-like phenotype and function. They may play a role in blood vessels maturation and remodeling^25^,^26^. In addition, they have been shown to secrete multiple angiogenic growth factors, such as hepatocyte growth factor (HGF) and vascular endothelial growth factor (VEGF)^27^,^28^. Considering the excellent osteogenic and angiogenic ability of both Sr-HT-Gahnite and ASCs, we hypothesize that an appropriate cell-biomaterial construct combining both elements may be a more effective approach for promoting both angiogenesis and osteogenesis.

## Results

### Cell spreading

**Figure 1:**
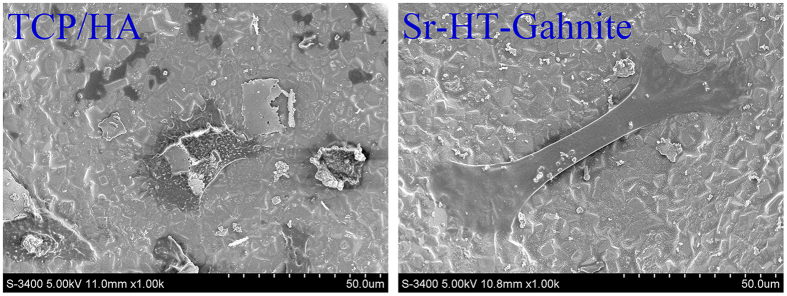
SEM observation of cell cultured on two scaffolds for 24 hours.

24 hours after cultured on the two materials, ASCs were observed and the results were illustrated in Fig. 1. Cells cultured on Sr-HT-Gahnite scaffolds appeared much flatter than those seeded on TCP/HA scaffolds.

### Cell proliferation

**Figure 2:**
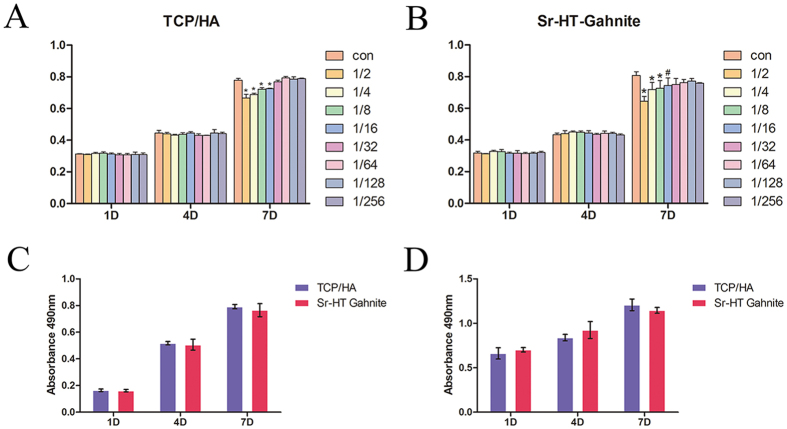
MTT assay of ASCs cultured in different concentrations of the TCP/HA (**A**) and Sr-HT-Gahnite dissolution products (**B**), in the optimal concentration (1/32 of 2 mg/mL) of dissolution products of the two materials (**C**), and on the two scaffolds (**D**).

ASCs were cultured with different concentrations of TCP/HA and Sr-HT-Gahnite dissolution products (1/258, 1/126, 1/64, 1/32, 1/16, 1/8, 1/4, 1/2 of 2 mg/mL) in osteogenic medium for 1, 4 and 7 days. As illustrated in Fig. 2A and B, the results of the MTT assay showed that both TCP/HA and Sr-HT-Gahnite exhibited no evidence of cytotoxicity. There was no obvious effect on metabolic activity in TCP/HA and Sr-HT-Gahnite groups compared to the blank control at day 1 and day 4. At day 7, higher concentrations of TCP/HA and Sr-HT-Gahnite dissolution products (1/16, 1/8, 1/4, 1/2 of 2 mg/mL) showed a decrease in cell metabolic activity. As shown in Fig. 2C, there was no obvious difference between TCP/HA and Sr-HT-Gahnite at the same concentration (1/32 of 2 mg/mL). There was no significant difference in total cell metabolic activity when ASCs were cultured on the two scaffolds as illustrated in Fig. 2D.

### Cell differentiation in dissolution products and on the scaffolds

**Figure 3:**
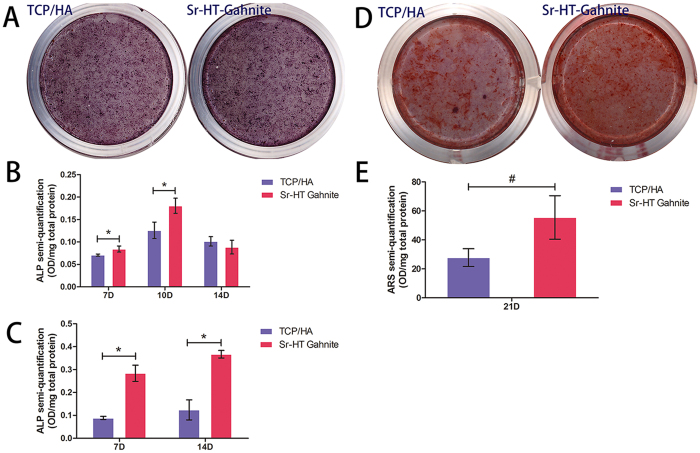
Osteogenic differentiation of ASCs. ALP staining was performed at day 10 (**A**), ALP semi-quantitative analysis was performed at day 7, 10, and 14 (**B**), and Alizarin Red S staining and semi-quantitative analysis was performed at day 21 (**D** and **E**) after cells were cultured in optimal dissolution products. ALP semi-quantitative analysis of cells cultured on the two scaffolds was performed at day 7 and 14 (**C**).

ASCs were cultured in TCP/HA and Sr-HT-Gahnite dissolution products at 1/32 dilution for the ALP activity assay. As shown in Fig. 3A, the cells treated with the Sr-HT-Gahnite dissolution products exhibited more pronounced ALP staining areas compared to the cells treated with the TCP/HA dissolution products. A semi-quantitative study was performed after 7, 10 and 14 days culture for dissolution products of TCP/HA and Sr-HT-Gahnite at 1/32 dilution. The results are shown in Fig. 3B. ALP activity of the Sr-HT-Gahnite dissolution products at day 7 and day 10 was significantly higher compared to the TCP/HA dissolution products. After 14 days culture, there was no distinct difference between two dissolution products at the 1/32 dilution. Semi-quantification analysis results from cells seeded on the two scaffolds are shown in Fig. 3C. ALP activity of ASCs at day 7 and day 14 was significantly enhanced on Sr-HT-Gahnite scaffolds compared to TCP/HA scaffolds. The results of Alizarin Red S staining were illustrated in Fig. 3D. Matrix mineralization was increased with Sr-HT-Gahnite dissolution products compared to TCP/HA dissolution products. The semi-quantitative analysis shown in Fig. 3E remained the same tendency the ARS staining findings. The ion concentrations of Ca, Si, Sr, Zn, and Al in TCP/HA and Sr-HT-Gahnite dissolution products are illustrated in Table 1. According to the ICP-AES data, only Ca and P ions were released from TCP/HA. In the dissolution product from Sr-HT-Gahnite particles, Ca, Si, Sr and Zn ions were examined and displayed. The Ca ion concentration in Sr-HT-Gahnite group was almost three times higher than that in TCP/HA group. There were none Al ion tested both in the dissolution products of TCP/HA and Sr-HT-Gahnite.

**Figure 4:**
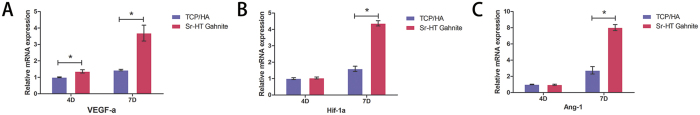
The expression of angiogenesis related genes: VEGF-α (**A**), HIF-1α (**B**) and ANG-1 (**C**) were measured by real-time PCR at day 4 and 7 after ASCs were cultured in optimal dissolution products of the two materials.

ASCs were cultured in TCP/HA and Sr-HT-Gahnite dissolution products at 1/32 dilution for 4 and 7 days to analyze the expression of angiogenic genes (Fig. 4). At day 4, the differences between TCP/HA group and Sr-HT-Gahnite group in the mRNA expression of hypoxia-inducible factor-1α (HIF-1α), and angiogenin-1 (ANG-1) was not significant, while the vascular endothelial growth factor-α (VEGF-α) mRNA expression of Sr-HT-Gahnite group was significantly enhanced compared to that of TCP/HA group. At day 7, expression of VEGF-α, HIF-1α, ANG-1, which reflect angiogenic differentiation, were promoted by the Sr-HT-Gahnite extracts compared to the TCP/HA dissolution products.

### Effects of dissolution products on HUVECs proliferation, migration, and differentiation

**Figure 5:**
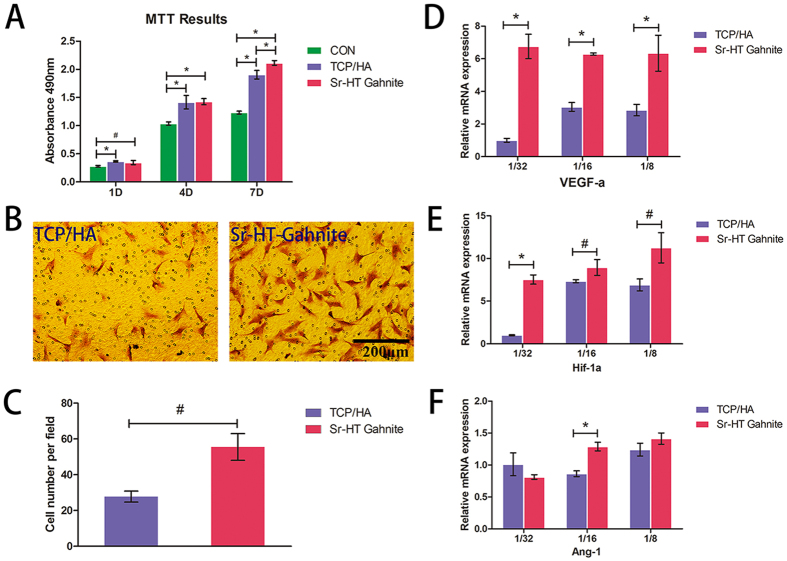
*In vitro* angiogenesis assay. MTT assay of HUVECs cultured in different concentration dissolution products of TCP/HA and Sr-HT-Gahnite (**A**); Transwell assay was conducted to detect HUVEC migration (**B** and **C**); The expression of angiogenic related genes: VEGF-α (**D**), HIF-1α (**E**) and ANG-1 (**F**) was measured by real-time PCR at day 4 after HUVECs were cultured in moderate dilutionsof dissolution products (1/8, 1/16 and 1/32) of the two materials.

HUVECs were cultured with TCP/HA and Sr-HT-Gahnite dissolution products at1/32 dilution for MTT assay and transwell assay. As shown in Fig. 5A, the results of MTT assay illustrated that both TCP/HA and Sr-HT-Gahnite increased metabolic activity of HUVECs compared to the blank control at day 1, 4 and 7. At day 1 and day 4, there was no obvious difference in the metabolic activity for HUVECs treated with TCP/HA and Sr-HT-Gahnite dissolution products. At day 7, Sr-HT-Gahnite dissolution products induced increased metabolic activity compared to the TCP/HA dissolution products. According to the results of transwell assay illustrated in Fig. 5B and C, HUVECs migration through transwell chambers significantly increased in response to Sr-HT-Gahnite dissolution products compared to TCP/HA dissolution products. After culturing HUVECs in the presence of different dilutions (1/32, 1/16 and 1/8) of TCP/HA and Sr-HT-Gahnite dissolution products for 4 day, angiogenesis related mRNA expression was tested. As shown in Fig. 5D–F, the mRNA expression levels of VEGF-α, HIF-1α were significantly increased by the Sr-HT-Gahnite dissolution products at different dilutions (1/32, 1/16 and 1/8) compared to TCP/HA dissolution products at the same concentration, while the mRNA expression levels of ANG-1 were promoted by the Sr-HT-Gahnite dissolution products at 1/16 dilution compared to TCP/HA dissolution products at the same concentration. There were no significant differences in expression between dissolution products of TCP/HA and Sr-HT-Gahnite at 1/8 and 1/32 dilution.

### *In vivo* study

#### Fluorochrome labeling histomorphometrical analysis

**Figure 6:**
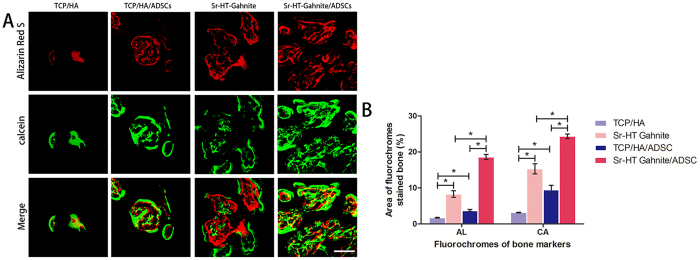
Sequential fluorescent labeling. The images in red and green indicated bone formation and mineralization at 4 and 6 weeks after operation, respectively (**A**); the percentage of AL and CA staining for each group was assessed by histomorphometric analysis (**B**). Scale bar = 500 μm.

Fluorescent labeling illustrates differences in newly formed bone area and mineralization at 4 and 6 weeks after surgery (Fig. 6A). The quantification data is provided in Fig. 6B. At week 4, the Sr-HT-gahnite (8.3 ± 0.9%) and Sr-HT-gahnite/ASCs scaffolds (18.6 ± 0.7%) showed a higher percentage of AL labeling area, as compared to TCP/HA (1.7 ± 0.1%) and TCP/HA/ASCs scaffolds (3.8 ± 0.3%). There was also a significant difference between TCP/HA and TCP/HA/ASCs scaffolds, and Sr-HT-gahnite and Sr-HT-gahnite/ASCs scaffolds respectively (p < 0.01). At week 6, the percentage of CA labeling in the group with Sr-HT-gahnite/ASCs scaffolds (24.4 ± 0.6%) was higher than that in groups with TCP/HA alone (3.2 ± 0.1%), TCP/HA/ASCs (9.5 ± 1.2%), and Sr-HT-gahnite alone (15.3 ± 1.4%). The percentage of CA labeling in the group with Sr-HT-gahnite alone was higher than those in TCP/HA (p < 0.01) and TCP/HA/ASCs (p < 0.01). The difference between TCP/HA and TCP/HA/ASCs was also significant (p < 0.01).

#### Histological analysis of bone regeneration

**Figure 7:**
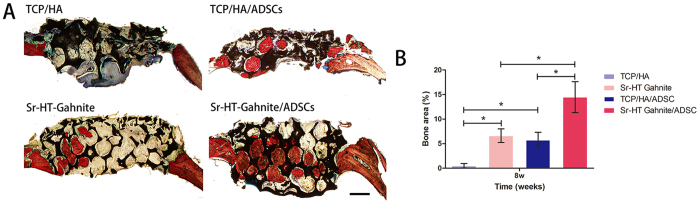
Histological images of newly formed bone in calvarial defects at 8 weeks post-operation (**A**); the percentage of new bone area was assessed by histomorphometric analysis (**B**). Scale bar = 1 mm.

The undecalcified specimens were stained with Van Gieson’s picro fuchsin for histological analysis of new bone formation. As shown in Fig. 7A, the newly formed bone almost filled the calvarial defect in Sr-HT-gahnite/ASCs scaffolds group. There was new bone tissue formed in the TCP/HA/ASCs and Sr-HT-gahnite scaffolds, but less compared to the Sr-HT-gahnite/ASCs scaffolds. In the TCP/HA group, there was little bone observed in the calvarial defect. The percentage of new bone area was 0.5 ± 0.5%, 6.6 ± 1.4%, 5.8 ± 1.5%, 14.5 ± 3.2% for the TCP/HA, TCP/HA/ASCs, Sr-HT-gahnite and Sr-HT-gahnite/ASCs, respectively (Fig. 7B).

**Figure 8:**
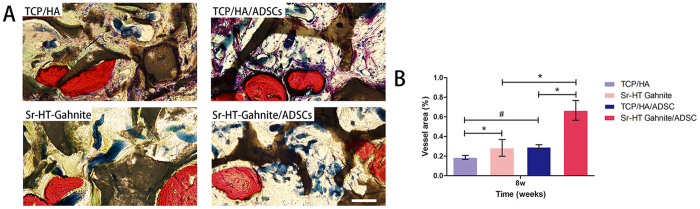
Histological images of newly formed blood vessels in calvarial defects (**A**); the percentage of newly formed blood vessel was assessed by histomorphometric analysis (**B**). Scale bar = 200 μm. (Notes: *P < 0.01, ^#^P < 0.05 versus control group).

For the evaluation of new blood vessels formation in calvarial defect area, the histological analysis of Microfil perfusion was performed. Blue spots from Microfil perfusion represented blood vessels. As shown in Fig. 8A and B, blood vessels could be observed in the TCP/HA group, but fewer blood vessels were formed compared to TCP/HA/ASCs group and Sr-HT-gahnite group. There were more newly formed blood vessels in Sr-HT-gahnite/ASCs group compared to the Sr-HT-gahnite group and the TCP/HA/ASCs group. The histomorphometric assay showed that the percentage of new vessel area was 1.9 ± 0.2‰, 2.8 ± 0.9‰, 3.0 ± 0.2‰, 6.7 ± 1.01‰ for the TCP/HA alone, TCP/HA/ASCs, Sr-HT-gahnite alone and Sr-HT-gahnite/ASCs group, respectively.

## Discussion

The use of stem cells has been designed to promote angiogenesis and osteogenesis for bone defect healing in tissue engineering approaches. As an abundant source of mesenchymal stem cells, adipose tissue became the focus of considerable interest in regenerative tissue engineering in recent decades. Due to its relatively high abundance and easy access, ASCs exhibit a potential advantage over other types of mesenchymal cells for clinical translation into regeneration therapies.

ASCs exhibit a strong capacity for *in vitro* expansion. A variety of approaches have been used to strengthen its multiple differentiation ability. In this study, we applied a scaffold, Sr-HT-Gahnite, to improve the osteogenic differentiation ability of ASCs and further enhance bone regeneration *in vivo*.

A variety of calcium silicate-based materials have been studied as promising bone substitutes. Bioactive trace elements such as Sr and Zn have been commonly utilized to improve the bioactivity and osteogenic differentiation ability of these materials^15^,^29^,^30^. As essential elements for the human body, calcium and silicon ions have been demonstrated to improve osteogenic differentiation of ASCs^31^,^32^. We assumed that the much higher concentration of Ca and Si ion released from Sr-HT-Gahnite attributed to its better osteogenic activity both *in vitro* and *in vivo*. The treatment of Sr ion could not only enhance the cell proliferation and osteogenic differentiation of ASCs *in vitro*^33^, but also accelerate new bone matrix formation *in vivo*^34^.

Zn is a crucial component of zinc finger-containing transcription factors. It plays important roles in bone development and mineralization^35^. Sr-HT-Gahnite is a mechanically strong and tough ceramic. It can be fabricated as a highly porous scaffold. As expected, the incorporation of Ca, Si, Sr, Zn ions in ceramic dissolution products improved the bioactivity and osteogenic differentiation of ASCs *in vitro* and their presence in scaffolds enhanced bone regeneration *in vivo*.

When ASCs were cultured on Sr-HT-Gahnite scaffolds, cells spread very well with more extensive spreading than seen with the control scaffold. The MTT assay reflects cells’ proliferative ability. There was no adverse effect on cell proliferation observed with Sr-HT-Gahnite scaffolds compared to TCP/HA scaffolds. When ASCs were cultured in the dissolution products of Sr-HT-Gahnite at day 1 and day 4, no decrease in cell proliferation occurred even in the highest dilution of 1/2. At day 7, higher dilutions from 1/2 to 1/16 showed a slight reduction in cell proliferation, while dilutions from 1/32 to 1/256 exhibited no adverse effect on cell metabolic activity. In addition, metabolic activity observed between the cells cultured in dissolution products at a dilution of 1/32 for either Sr-HT-Gahnite or clinically used TCP/HA showed no significant difference. This phenomenon confirmed the acceptable biocompatibility of Sr-HT-Gahnite. The ALP activity analysis results from cells cultured in dissolution products or on scaffolds suggested that the osteogenic differentiation ability of ASCs were enhanced by Sr-HT-Gahnite treatment. The ARS staining and semi-quantification of cells cultured in dissolution products of Sr-HT-Gahnite further confirmed its osteogenic activity *in vitro* study.

For *in vivo* study, a critical-sized calvarial bone defect model in rats was used to assess the potential effect of Sr-HT-gahnite/ASCs complex on promoting vascularized bone formation. According to the results of histological analysis of bone regeneration *in vivo*, there was little new bone observed in the TCP/HA group, while more newly formed bone was observed in the Sr-HT-Gahnite group. When Sr-HT-Gahnite and TCP/HA scaffolds were seeded with ASCs, there was obviously more bone tissue formed, especially in the Sr-HT-Gahnite/ASCs group. ASCs are known for their ability to accelerate new bone formation and promote angiogenesis by secreting angiogenic factors. Containing ions including Ca, Si, and Sr, Sr-HT-Gahnite scaffolds have been confirmed to be beneficial to angiogenesis. Angiogenesis and neo-vascularization are important for cell survival, integration and regeneration of new bone tissue. In the absence of ASCs, more newly formed blood vessels were achieved for the Sr-HT-Gahnite group as compared with the control TCP/HA group *in vivo*. When seeded with cells, the amount of newly formed blood vessels was promoted in both the Sr-HT-Gahnite/ASCs group and the TCP/HA/ASCs group.

In the present study, the dissolution products of Sr-HT-Gahnite up-regulated the expressions of VEGF-α, HIF-1α and ANG-1 by ASCs *in vitro*, which indicated the stimulatory effect of Sr-HT-Gahnite on the angiogenic activity of ASCs as we expected. VEGF regulates all the critical steps of angiogenesis related processes including endothelial cell proliferation, migration and tube formation. It has been known as a key angiogenic factor for its crucial role in enhancing blood vessel formation. In this study, the dissolution products of Sr-HT-Gahnite and TCP/HA could both stimulate the proliferation of HUVECs, however, the stimulatory effect of Sr-HT-Gahnite was higher than that of TCP/HA. In addition, HUVECs migration across the membrane of a transwell was increased in Sr-HT-Gahnite group, indicating its superior ability to promote cell migration. Moreover, the mRNA expression of VEGF-α and HIF-1α were up-regulated when HUVECs were cultured in the dissolution products at dilutions of 1/8, 1/16 and 1/32, and the mRNA expression of ANG-1 was up-regulated at the dilution of 1/16. All of these results indicated that the *in vitro* induction of angiogenesis was enhanced by dissolution products of Sr-HT-Gahnite, which probably can be attributed to the incorporation of Ca, Si and Sr ions. According to our results, we assumed that Sr-HT-Gahnite alone can recruit host vein endothelial cells (VECs). In the absence of ASCs, it could promote angiogenesis and finally facilitate bone regeneration. Combined with ASCs, Sr-HT-Gahnite not only enhances the osteogenesis of implanted ASCs, but also could promote angiogenesis, thereby achieving optimal effects in the bone regeneration of calvarial defects.

## Conclusions

In summary, being a strong, highly porous and biocompatible bio-ceramic, Sr-HT-Gahnite showed no adverse effect on cell viability. Sr-HT-Gahnite not only promoted osteogenic and angiogenic differentiation of ASCs, but also enhanced the proliferation, migration and expression of angiogenic factors by HUVECs. Moreover, in a rat critical-sized calvarial defect model, the combination of Sr-HT-Gahnite and ASCs enhanced both osteogenesis and angiogenesis *in vivo*. Our study suggests that through incorporation of Ca, Si, Sr, and Zn the Sr-HT-Gahnite scaffolds could act as cell carrier for ASCs for the construction of vascularized tissue-engineered bone.

## Materials and Methods

### Specimen preparation

TCP/HA and Sr-HT-Gahnite powders were prepared according to previously published procedures. The TCP/HA and Sr-HT-Gahnite scaffolds were fabricated by a polymer sponge replication technique^19^,^36^.

### Preparation of biomaterial dissolution products

Prior to prepare dissolution products, TCP/HA and Sr-HT-Gahnite agglomerates were ground and sieved to a median size (D50) of 2 micron. The dissolution products of TCP/HA and Sr-HT-Gahnite particles were prepared and sterilized. Two grams of each kind of particles was soaked in 10 mL osteogenic differentiation medium (growth medium supplemented with 50 mg/ml L-ascorbic acid, 10 mM glycerophosphate, and 100 nM dexamethasone)^37^. After 24 hours incubation, the resultant solution was obtained by centrifuging paritcles and media. The solution was sterilized through a 0.22 μm filter (Merck Millipore, Darmstadt, Germany) for the following experiments. The concentrations of Ca, Si, Sr, Zn and Al in TCP/HA and Sr-HT-Gahnite dissolution products were examined by inductively coupled plasma atomic emission spectroscopy (ICP-AES, IRIS 1000, Thermo Elimental).

### Culture of ASCs on the different scaffolds

Accroding to previously published procedures^23^, ASCs were isolated from the inguinal fat pad of SD rats via 1 mg/mL collagenase digestion. The subcutaneous adipose tissue was minced, then washed with phosphate balanced solution (PBS) and digested at 37 °C while shaking at 80 rpm for 60 min.

The digested fat tissue was centrifuged at 1800 rpm for 10 min. Then, it was filtered through a 40 micron cell strainer to remove undigested tissue. Cells were resuspended in growth medium (Dulbecco’s Modified Eagle Media, DMEM) supplemented with 10% fetal bovine serum (FBS, Thermal Fisher Scientific, Waltham, USA). After 2 days’ culture, culture medium was changed to remove non-adherent cells. After the primary passage, cells were incubated in the osteogenic medium. Cell lines used for the following study were in passage 2 to 3.

#### Cell morphology

Scaffolds were sterilized by autoclave prior to use in experiments. ASCs at a density of 2.0 × 10^5^ cells per scaffold in 100 uL cell suspension were seeded on the scaffolds for cell morphology assay. After 24 hours of culture, they were fixed in 2.5% glutaraldehyde. The specimens were dehydrated in graded series of ethanol and dried in hexamethyldisilazane. Then, the scaffolds were sputter-coated with gold and examined by a Magellan 400 field-emission scanning electron microscope (FEI, Hillsboro, OR, USA).

#### Metabolic activity

ASCs were cultured on the two types of scaffolds placed in 24-well plates for up to 7 days. After 1, 4 and 7 days of culture, a MTT [3-(4, 5-dimethylthiazol-2-yl)-2, 5-diphenyl tetrazolium bromide] assay was carried out to examine the total metabolic activity of cells. Briefly, specimens were washed with PBS three times, then, they were transferred to a new 24-well plate. 200 uL of culture medium supplemented with 20 uL 5 mg/mL of MTT solution was added to each targeted well. After incubation for a further 4 hours, the culture medium was carefully replaced with 200 uL of dimethyl sulfoxide (DMSO) and placed on a rotating tray for 15 min. Then, the supernatent was transferred to a 96-well plate. The absorbance was measured at 490 nm by use of ELX Ultra Microplate Reader (Bio-tek, Winooski, VT, USA).

#### Cell differentiation

ASCs differentiation on TCP/HA and Sr-HT Gahnite was assessed using a semi-quantitative analysis of alkaline phosphatase (ALP) activity. After 7 and 14 days of cells culture on the two types of the scaffolds, the samples were washed with PBS and incubated with p-nitrophenyl phosphate (Sigma) for 4 hours. The optical density values for absorbance at 405 nm (Bio-tek) were measured to determine ALP activity. The intracellular total protein content was measured by use of the microBCA protein assay kit (Thermo Fisher Scientific, Waltham, MA, USA) according to the manufacturer’s instructions. The ALP quantity analyses were normalized to the total protein content.

### Effect of bio-ceramic dissolution products on ASCs

To investigate the total cell viability of ASCs in response to various concentrations of TCP/HA and Sr-HT Gahnite dissolution products, MTT assay was carried out. ASCs were incubated in 96-well plates in triplicate at a density of 4 × 10^3^ cells per well for 4 hours. Then, the culture medium was removed and the osteogenic medium supplemented with various concentrations of the dissolution products was filled in. After incubation for an additional 1, 4 and 7 days, total cell metabolic activity was measured as described above in section “Metabolic activity”.

Alkaline phosphatase activity and calcium deposition assays were conducted to test the effects of different concentrations TCP/HA and Sr-HT Gahnite dissolution products on the osteogenic differentiation of ASCs. Cells were cultured in 24-well plates in osteogenic medium with dissolution products of TCP/HA and Sr-HT-Gahnite. ALP staining was performed by use of an ALP kit (Beyotime Biotechnology, Shanghai, People’s Republic of China) after 10 days of culture. ALP semi-quantitative analysis was performed at 7, 10 and 14 days. After 21 days of incubation, Alizarin Red S staining was conducted to observe calcium deposition. Briefly, cells were treated with 95% alcohol for fixation and washed with distrilled water. Cells were stained with 0.5% Alizarin Red S staining solution for mineralization examination. After washing several times with distilled water, specimens were observed. For the quantification analysis, the stained samples were desorbed using 10% cetylpyridinium chloride (Sigma). Absorbance values at 590 nm were recorded. Intracellular total protein content was determined by use of the microBCA protein assay kit (Thermo). ALP and calcium deposition quantity analyses were normalized to total protein content.

Angiogenic differentiation of ASCs was determined in cells cultured in the different extracts for 4 and 7 days. Total RNA was extracted by use of trizol reagent. Complementary DNA (cDNA) was synthesized by use of the primescript^TM^ RT reagent kit (Takara, Bio Inc, Otsu, Japan). Primers for the selected angiogenesis related genes, including VEGF-α, Hif-1α, and ANG-1 were the same as those described in previously studies^38^,^39^. Expressions of these genes were quantified by use of Real-time PCR with SYBR Premix Ex Taq II (Takara). The relative expression levels for each gene of interest were normalized to that of the housekeeping gene GAPDH.

### Effect of different bio-ceramic dissolution products on *in vitro* angiogenesis of HUVECs

A moderate dilution ratio of 1/32 of dissolution products was applied to examine the stimulatory effect of different extracts of TCP/HA and Sr-HT-Gahnite on HUVECs proliferation. The HUVECs were cultured in 96-well plates at a density of 3 × 10^3^ cells per well. After 24 hours of incubation, the culture medium was removed. The TCP/HA and Sr-HT-Gahnite dissolution products diluted with DMEM containing 10% FBS at different concentrations were added to target wells. Cells cultured with DMEM containing 10% FBS were used for the control group. At day 1, 4 and 7 of culture, the MTT assay was conducted by use of a standard procedure as described above in section “Metabolic activity”.

HUVECs were seeded into 12-well plates at a density of 4 × 10^4^ cells per well. After the culture medium was replaced by the TCP/HA and Sr-HT-Gahnite dissolution products diluted with DMEM containing 10% FBS at different concentrations 1/8, 1/16 and 1/32, the cells were cultured for another 4 days for RNA extraction. Expression of angiogenesis related genes VEGF-α, HIF-1α, and ANG-1 were evaluated by use of Real-time PCR with SYBR Premix Ex Taq II (Takara). The relative expression levels for each gene of interest were normalized to that of the housekeeping gene GAPDH as described above.

To test the migratory ability of HUVECs treated with the Sr-HT Gahnite and TCP/HA dissolution products, transwell plates with a pore size of 8 μm (Millipore Inc, Billerica, MA) were used to perform transmigration assays. Firstly, cells were pre-cultured in dissolution products of either TCP/HA or Sr-HT-Gahnite at 1/32 dilution for 7 days followed by seeding 4 × 10^4^ HUVECs cells in DMEM containing 10% FBS in the upper chamber^40^. The lower chamber was added with the dissolution products of either TCP/HA or Sr-HT-Gahnite at 1/32 dilution supplemented with 10% FBS. After 18 hours of incubation, the upper side of the membrane was carefully wiped with cotton swab to remove non-migrating cells.

Cells that had traversed the membrane were fixed and stained with 0.1% crystal violet. They were washed in PBS for several times and observed. The cells numbers in five random fields of each sample were counted.

### *In vivo* implantation

For *in vivo* experiments, 12-week-old male SD rats were used in this study. All animals were obtained from the Ninth People’s Hospital Animal Center (Shanghai, China). The experimental protocol was approved by the Animal Care and Experimnt Committee of the Ninth People’s Hospital, and the methods were performed in accordance with the approved guidelines. A critical-sized calvarial defect model was performed according to our previous study^41^. The animals were anesthetized with intraperitoneal injection of pentobarbital (Nembutal 3.5 mg/100 g). Two circular 5-mm diameter calvarial defects were prepared by use of a trephine bur (ITI Dental Implant System; Straumann). A total of 32 defects from 16 SD rats were randomly filled with the following four groups: (A) TCP/HA only (n = 8); (B) Sr-HT-Gahnite only (n = 8); (C) TCP/HA and ASCs (n = 8); and (D) Sr-HT-Gahnite and ASCs (n = 8). The wound was closed in layers by use of 4–0 resorbable sutures.

### Sequential fluorescent labeling

The new bone formation and mineralization were labeled by use of a polychrome sequential fluorescent labeling as previously described^22^,^42^. The animals were intraperitoneally injected with 30 mg/kg of alizarin red (AL, Sigma) and 20 mg/kg of calcein (CA, Sigma) at 4 and 6 weeks post-operation.

### Microfil perfusion

At 8 weeks after surgery, rats were euthanized and perfused with Microfil (Flowtech, USA) for the evaluation of blood vessel formation. A long incision was made from the front limbs down to the xyphoid process^43^. Then, one side of the sternum was cut with scissors, and the rib cage was retracted. After clamping the descending aorta, the left ventricle was penetrated with an angiocatheter. The inferior vena cava was incised and heparinized saline was perfused. Subsequently, 20 mL of Microfil was perfused.

### Histological analysis

The whole calvarial samples were dehydrated in an ascending series of alcohols and embedded in polymethylmethacrylate (PMMA). The specimens were sagittally cut into 150-mm-thick sections by use of a microtome (Leica TCS, Germany), ground and polished to a thickness of 40–50 μm. The samples were observed for fluorescent labeling by use of a confocal laser scanning microscope (CLSM, Leica TCS, Germany).The fluorochrome staining results was quantified by use of a personal computer-based image analysis system (Image-Pro Plus, Media Cybernetics, USA), as previously described^44^. The excitation/emission wavelengths used to visualize each of the fluorophores were 543/617 nm (AL, red) and 488/517 nm (CA, green). Red (AL), and green (CA) areas represent the new bone formation and mineralization at 4 and 6 weeks post operation, respectively. For histological observation, polished sections were stained with Van Gieson’s picro fuchsin. The area of new bone was expressed as the percentage of the newly formed bone area to the entire calvarial defect area. Moreover, each blue spot from the Microfil perfusion indicated a new blood vessel. The areas of new bone in the stained samples and the area of blue spots (vessel area) for each group were quantitatively evaluated using Image Pro 5.0.

### Statistical analysis

Analysis for differences between TCP/HA group and Sr-HT-Gahnite group and among the various groups was performed by use of T-tests assuming equal variances and one-way analysis of variance with Student-Newman-Keuls post-hoc test based on the normal distribution. All statistical analyses were carried out by use of the SPSS 19.0 statistical software package. Results are presented as mean ± standard deviation. Differences were considered significant for P < 0.05 and P < 0.01.

## Additional Information

**How to cite this article**: Wang, G. *et al*. Effects of Sr-HT-Gahnite on osteogenesis and angiogenesis by adipose derived stem cells for critical-sized calvarial defect repair. *Sci. Rep.* **7**, 41135; doi: 10.1038/srep41135 (2017).

**Publisher's note:** Springer Nature remains neutral with regard to jurisdictional claims in published maps and institutional affiliations.

## Figures and Tables

**Table 1:** Ion concentrations of dissolution products from TCP/HA and Sr-HT-Gahnite.

Sample	Ion concentration (mg/L)					
	Ca	P	Si	Sr	Zn	Al
TCP/HA	9.5	28.8	—	—	—	<2
Sr-HT-Gahnite	29.8	—	53	7.7	9.8	<2
